# Epstein-Barr Virus-Associated Aggressive Natural Killer Cell Leukemia: Challenges and Emerging Therapies

**DOI:** 10.7759/cureus.66338

**Published:** 2024-08-06

**Authors:** Ahmad Hamdan, Chun Chou, Daniel Rust, Andrew Strand

**Affiliations:** 1 Infectious Diseases, Tufts Medical Center, Boston, USA; 2 Internal Medicine, University of Pennsylvania, Philadelphia, USA; 3 Pathology, Tufts Medical Center, Boston, USA; 4 Infectious Disease, Tufts Medical Center, Boston, USA

**Keywords:** hemophagocytic lymphohistiocytosis (hlh), antiviral drugs, late acute liver injury, chronic active epstein-barr virus (caebv), aggressive natural killer cell leukemias, ebv positive lymphoproliferative disorder

## Abstract

A 24-year-old Ecuadorian female, previously diagnosed with acute fatty liver (AFL) during pregnancy, developed constitutional symptoms, jaundice, and abdominal pain in a subsequent pregnancy, prompting investigations that suggested a recurrence of AFL. She underwent an elective abortion, which resulted in the resolution of her abdominal pain, and a liver biopsy, which showed granulomatous inflammation and lymphocytic infiltration. She later presented with abdominal distention, productive cough, and persistent constitutional symptoms and jaundice. Extensive laboratory and imaging studies indicated sepsis, acute liver injury, and disseminated intravascular coagulopathy. Her serum Epstein-Barr virus (EBV) level was elevated. Special staining of her previous liver biopsy revealed EBV-positive natural killer (NK) cells. A bone marrow biopsy also revealed EBV-positive NK cells. She was diagnosed with aggressive NK cell leukemia (ANKL) with or without chronic active EBV (CAEBV). Treatment included dexamethasone, atovaquone, bortezomib, and ganciclovir, with plans for a stem cell transplant. However, her course was complicated by infections and multi-organ failure, resulting in her passing. This case highlights the rarity and challenges in managing EBV-associated ANKL, emphasizing the need for early detection and improved treatment options, with stem cell transplantation offering the best prognosis.

## Introduction

Epstein-Barr virus (EBV) is a common virus with a high global infection rate. Primary infection is often asymptomatic or results in mild symptoms, after which an asymptomatic, lifelong carrier state is established and maintained by immune surveillance [1,2]. However, in rare cases, EBV can lead to malignancies and lymphoproliferative disorders (LPD), often related to immune impairment, genetic predisposition, and environmental factors [3,4]. LPD can affect B cells, T cells, or natural killer (NK) cells [3]. EBV-associated T/NK cell LPD are categorized into six types, including chronic active EBV (CAEBV) and aggressive NK cell leukemia (ANKL) [5].

Treatment options for T/NK cell LPD are limited and primarily rely on anecdotal evidence and small studies. To date, no standardized treatment protocol is available. Chemoradiation and stem cell transplantation are the only treatments that have been shown to improve survival [6]. Recent research suggests potential benefits from JAK1 and JAK2 inhibitors like ruxolitinib, as well as PD-1/PD-L1 antibodies, such as the immune checkpoint inhibitor pembrolizumab [7,8]. Additionally, a combination of ganciclovir and either histone deacetylase inhibitors or bortezomib has shown some potential benefit [9].

## Case presentation

A 24-year-old female from Ecuador, G2P0111, with a history of presumed acute fatty liver (AFL) complicating her first pregnancy a year prior and leading to induced preterm birth, presented with several symptoms. Her symptoms began five months ago when she became pregnant again and developed jaundice, weight loss, poor appetite, and decreased oral intake. One month ago, she developed right upper quadrant (RUQ) abdominal pain and was found to have elevated liver function tests (LFTs). She was diagnosed again with AFL, underwent an elective abortion by dilation and curettage, and was started on ursodiol. A liver biopsy at the time showed lobular granulomatous inflammation, macrovesicular steatosis, hepatocellular cholestasis, perivenular and pericellular fibrosis, and lymphocytic infiltrate of portal tracts with a negative iron stain. Although her abdominal pain resolved, her other symptoms persisted. She is now presenting with two weeks of worsening abdominal distention and one week of productive cough with yellow phlegm, diarrhea, labial swelling, and dysuria, in addition to ongoing jaundice, weight loss, poor appetite, and decreased oral intake.

Her first pregnancy was complicated by elevated LFTs and thrombocytopenia presumed to be secondary to AFL of pregnancy resulting in induced preterm birth at seven months of gestation. At the time, investigations included a positive serum antinuclear antibody (ANA) titer (1:160) with negative other autoimmune markers. The serum iron panel was not suggestive of hemochromatosis. Serum hepatitis A, B, C, and E, Coxiella, Leptospira, Brucella, and Bartonella serologies were negative. Additionally, serum human immunodeficiency virus (HIV) antigen (Ag) and antibody (Ab) and EBV viral capsid antigen immunoglobulin (Ig) M were negative. Serum cytomegalovirus (CMV), herpes simplex virus, and varicella zoster virus polymerase chain reaction (PCR) were also negative. RUQ ultrasound (US) showed increased echogenicity of liver parenchyma likely representing fatty infiltration. Magnetic resonance cholangiopancreatography did not show any intra-hepatic lesions or biliary obstruction/dilation. Her LFTs improved but did not completely return to normal in the postpartum period.

She was born in Quito, Ecuador, and immigrated to Massachusetts at the age of 17 years. As a child, she was exposed to chickens and consumed unpasteurized dairy products. Her family history was unremarkable. She had no drug allergies. Her only home medication was ursodiol, and she did not take any over-the-counter medications or herbal supplements. She denied the use of tobacco, alcohol, or other drugs. Her physical examination was notable for normal blood pressure, tachycardia (125 beats/minute), cachexia, scleral icterus, jaundice, abdominal distention with a fluid wave, and anasarca. 

Investigations on admission were remarkable for white blood cell (WBC) count 19 k/μL (reference {ref}: 4-11 k/μ), lymphocytes 83% (ref: 20-40%), hemoglobin 9.3 g/dL (ref: 11-16 g/dL), platelets 73 k/μL (ref: 150-400 k/μL), sodium 129 mmol/L (ref: 135-146 mmol/L), bicarbonate 12 mmol/L (ref: 20-32 mmol/L), anion gap 8 mmol/L (ref: 3-14 mmol/L), aspartate transaminase 244 U/L (ref: 6-42 U/L), alanine transaminase 118 U/L (ref: 0-55 U/L), alkaline phosphatase 646 U/L (ref: 30-130 U/L), total bilirubin 10.3 mg/dL (ref: 0.2-1.2 mg/dL), direct bilirubin 7.3 mg/dL (ref: 0-0.5 mg/dL), INR 2, albumin 1 g/dL (ref: 3.2-5 g/dL), fibrinogen <35 mg/dL (ref: 170-440 mg/dL), lactate dehydrogenase (LDH) 1141 U/L (ref: 110-250 U/L), and human chorionic gonadotropin level negative (ref <5 mIU/mL). Serum acetaminophen, alcohol, and salicylate levels and urine drug screen were negative. Her urinalysis was negative for WBCs, protein, or blood. A RUQ US showed hepatomegaly and ascites. Computed tomography of the chest, abdomen, and pelvis showed patchy ground glass opacities, tree-in-bud nodularity, right hilar adenopathy, bilateral pleural effusions, hepatic steatosis, and hepatosplenomegaly. A paracentesis was performed showing a WBC count of 39 cells/µL (ref <500 cells/µL), neutrophils of 1 cell/µL (ref: <250 cell/µL), and a serum-ascites album gradient of 1.2 g/dL. Blood and peritoneal fluid cultures were negative.

She was admitted with sepsis of unclear source, acute liver injury, and possible disseminated intravascular coagulopathy (DIC). The gastroenterology team recommended a workup for autoimmune liver diseases, which included a nonreactive serum ANA and normal levels of serum alpha-1 antitrypsin, ceruloplasmin, copper, tissue transglutaminase IgA, and IgG subclasses. Additionally, serum anticardiolipin Ab, anti-smooth muscle Ab, anti-mitochondrial Ab, anti-double-stranded deoxyribonucleic acid (DNA) Ab, and beta-2 glycoprotein were negative. Iron studies were abnormal but not suggestive of hemochromatosis. Hematology recommended transfusions per protocol as coagulopathy was thought to be from liver disease. The patient later developed a fever of 38°C, and an infectious diseases consultation was requested. Given the granulomatous changes in the liver and changes in lungs, the differential diagnosis was broad and included mycobacteria, brucella, bartonella, treponema, histoplasma, cryptococcus, EBV, and CMV, among others. She was started on intravenous (IV) ceftriaxone 1 g daily and oral doxycycline 100 mg twice daily for community-acquired pneumonia for five days.

Infectious diseases workup included a sputum culture that grew normal flora. Sputum acid-fast bacilli smear and culture were negative x3. Stool gastrointestinal (GI) pathogen panel by PCR and *Clostridium difficile *enzyme immunoassay were negative. Urine and vaginal *Neisseria gonorrhoeae* and *Chlamydia trachomatis* nucleic acid amplification test and urine histoplasma antigen were also negative. Serum interferon release assay, beta-d-glucan, galactomannan, HIV Ag/Ab, and serologies for hepatitis A, B, C, E, syphilis, *Bartonella henselae*, *Bartonella quintana*, Brucella, and Coccidioides were negative as well. Serum CMV PCR was negative but EBV PCR was >2 million copies/mL.

**Figure 1:**
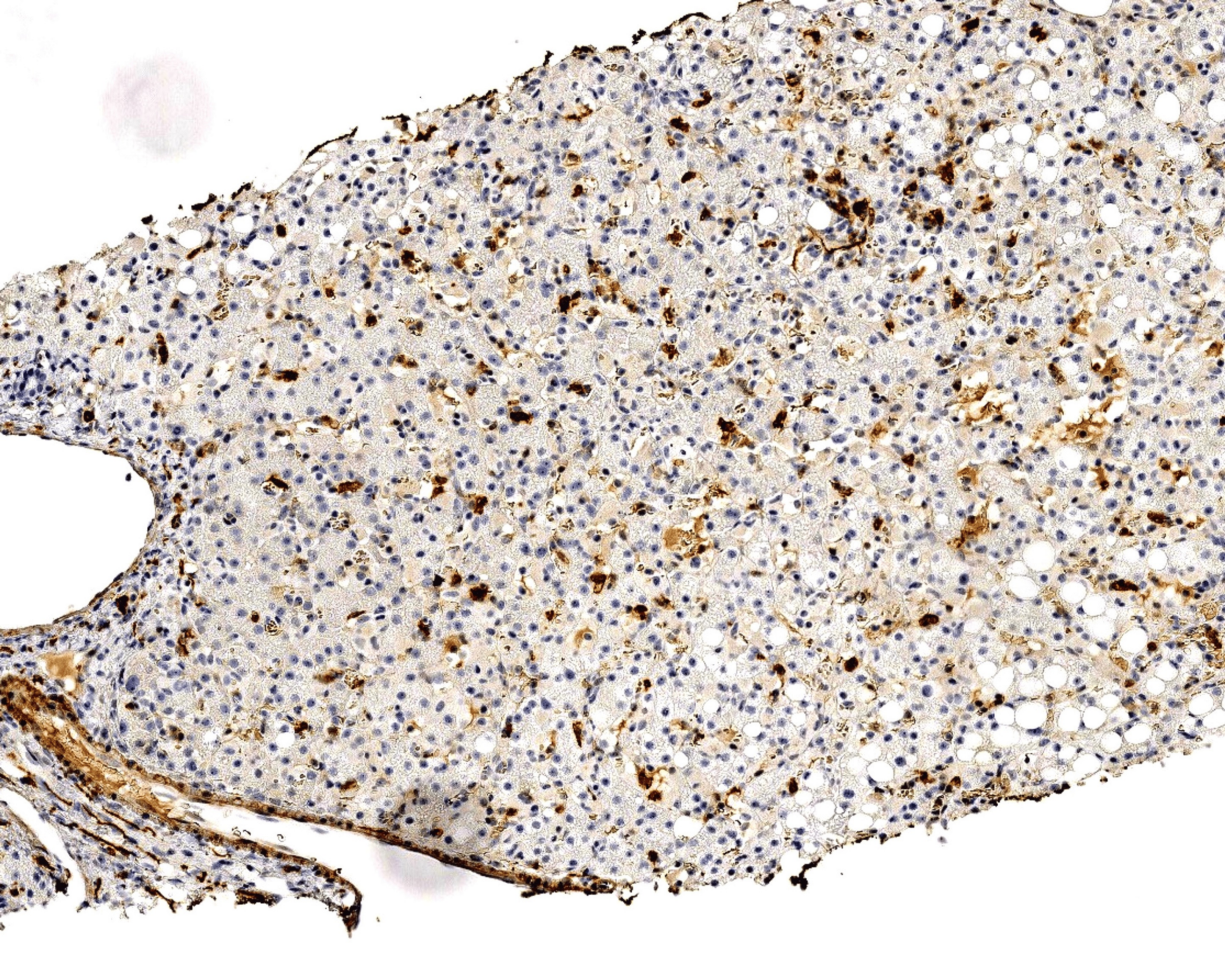
Liver biopsy immunohistochemical stain for CD56 (100x) highlights frequent NK cells present in scattered distribution and small aggregates. NK: natural killer

**Figure 2:**
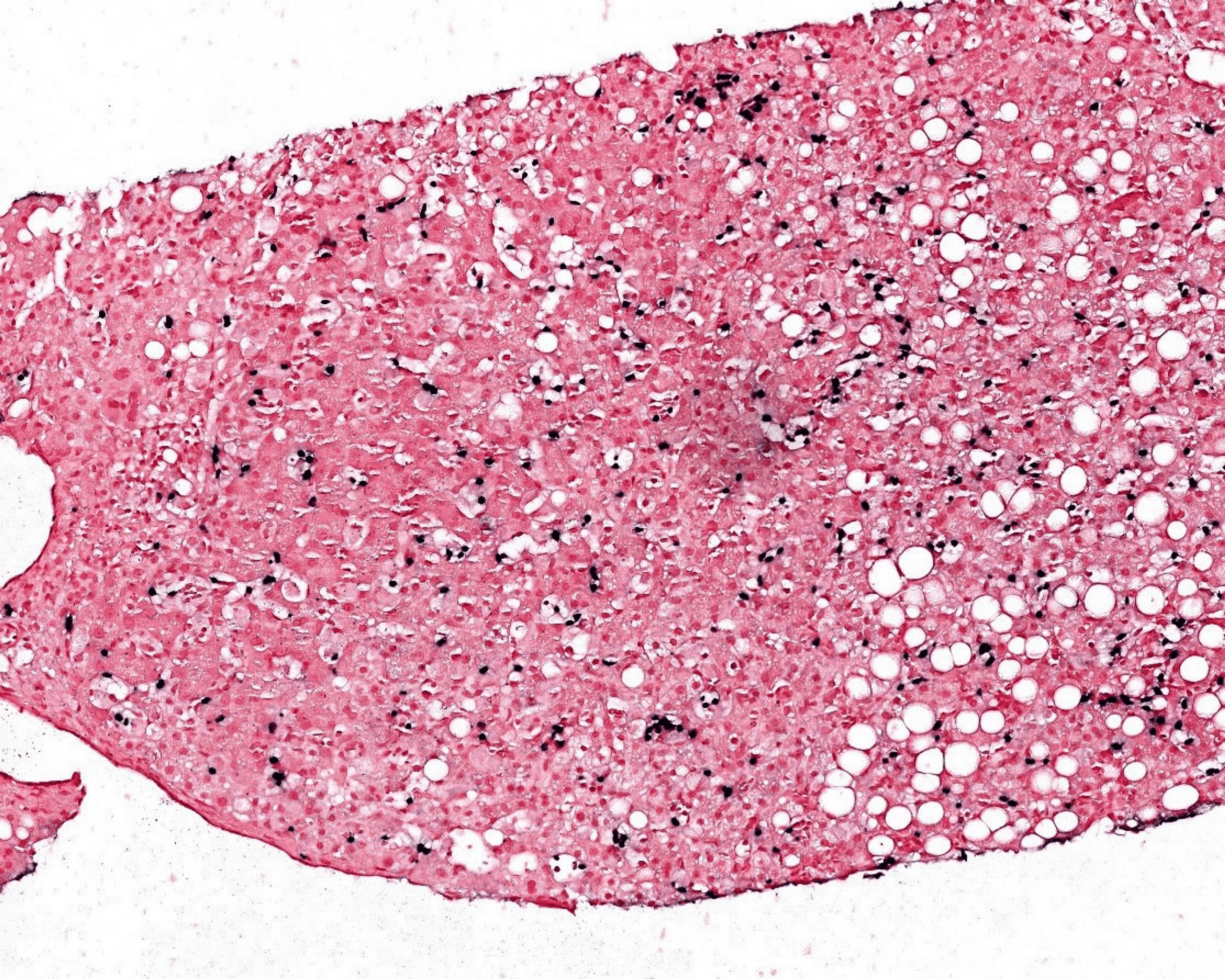
Liver biopsy in situ hybridization for EBV (100x) highlights cells in a similar scattered distribution and in small aggregates. The findings support the presence of an EBV-positive NK cell population. EBV: Epstein-Barr virus; NK: natural killer

Given this finding, immunohistochemical stains were requested on the liver biopsy, which was done a month prior, and revealed EBV-positive NK cells suggesting ANKL or CAEBV (Figures 1, 2). Peripheral blood flow cytometry demonstrated an expanded NK cell population.

**Figure 3:**
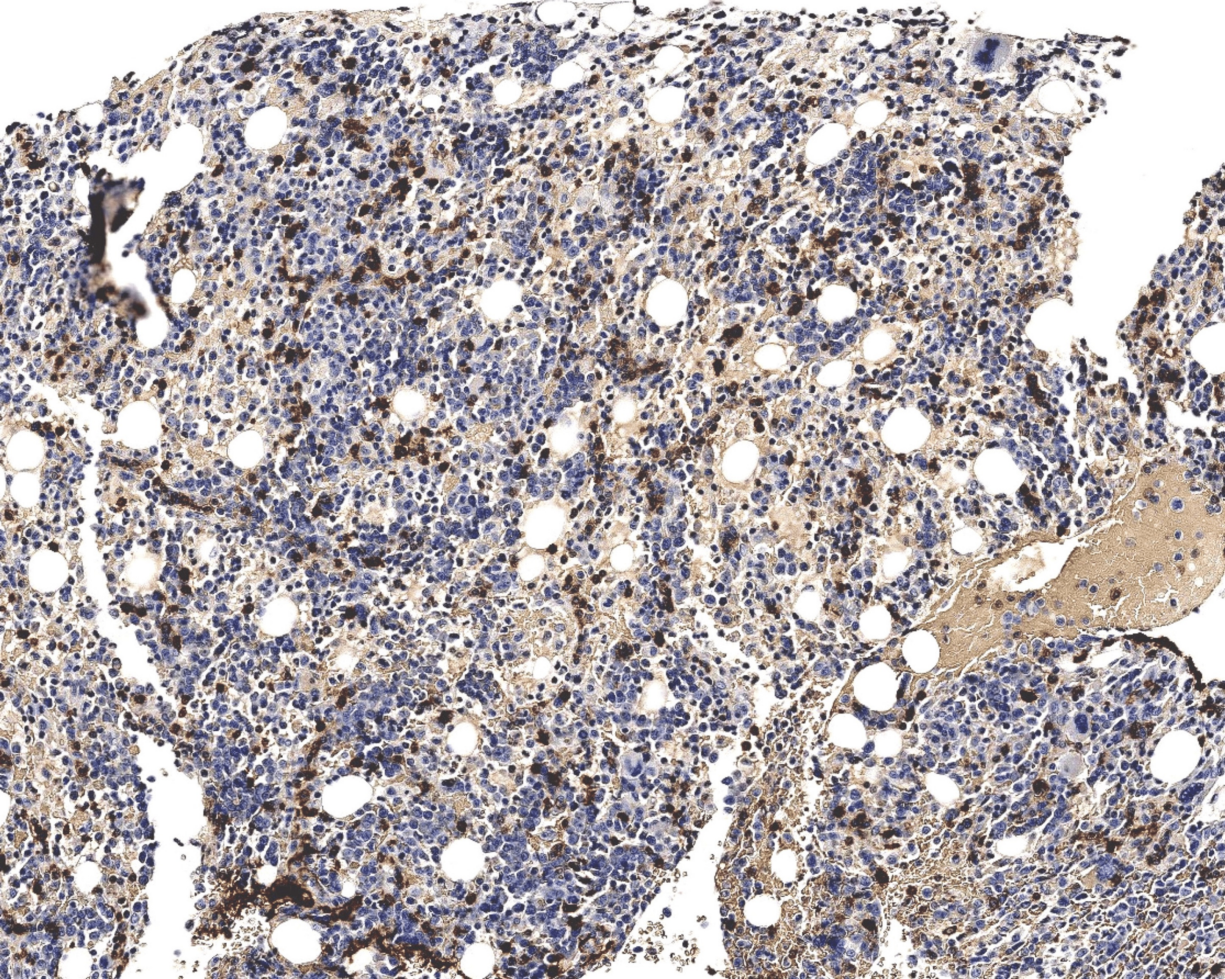
Bone marrow trephine biopsy immunohistochemical stain for CD56 (100x) highlights a CD56+ NK cell population. NK: natural killer

**Figure 4:**
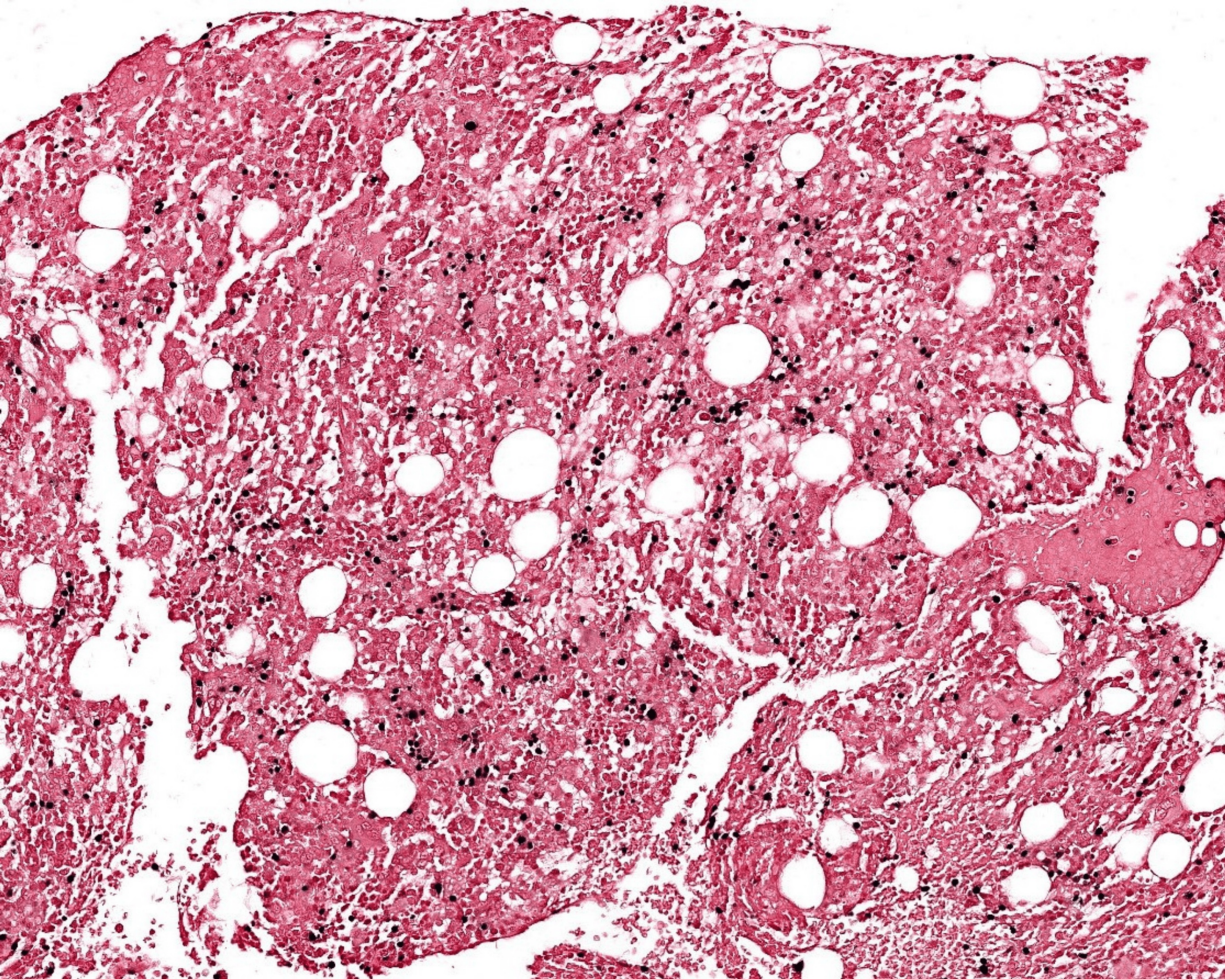
Bone biopsy in situ hybridization for EBV (100x) highlights cells in similar distribution. The findings support bone marrow involvement by EBV-positive NK cell proliferation. EBV: Epstein-Barr virus; NK: natural killer

The patient then underwent a bone marrow biopsy which showed involvement by EBV-positive abnormal NK cell population representing 78% of cellularity and hemophagocytosis (Figures 3, 4). Trisomy 8 and deletion 11q were identified but no aneuploidy was detected by fluorescence in situ hybridization. Markers included positive CD56, CD16, CD8, CD38, dim CD52, and HLA-DR and negative CD3, CD57, CD5, CD7, CD14, CD25, TCR alpha, TCR beta, TCR gamma, TCR delta, B cell markers, and myeloid markers. Abnormal immunophenotypic features of the NK cell population included CD7 negativity, CD57 negativity, and strong CD8 positivity.

The favored diagnosis was ANKL with or without CAEBV of NK cell type (given prior liver injury, liver NK cell infiltration, and elevated EBV viral load) and hemophagocytic lymphohistiocytosis (HLH).

The patient was started on oral dexamethasone 20 mg daily for HLH and oral atovaquone 1500 mg daily for *Pneumocystis jirovecii* pneumonia prophylaxis and later, on IV bortezomib 0.7 mg/m^2^ (on days one, four, eight, 11) and IV ganciclovir 5 mg/kg twice daily. Serum EBV level trended down to 799,792 copies/mL on completion of the bortezomib. A week after, serum EBV level increased to 1,489,873 copies/mL. Alemtuzumab was later started for NK leukemia-directed therapy. Serum EBV level eventually trended down to 429,343 copies/mL. Allogeneic stem cell transplant was the ultimate goal of therapy.

Her course, however, was complicated by *Escherichia coli *and *Candida albicans *right labial skin and soft tissue infection requiring incision and drainage as well as encephalopathy, acute hypoxic respiratory failure related to epistaxis and oropharyngeal bleeding, hemorrhagic shock in the setting of worsening DIC and GI bleed, abdominal compartment syndrome, and streptococcal mitis/oralis bacteremia. After the goals of care discussion, the patient was made comfort measures only and passed away.

## Discussion

Here, we presented a rare yet highly morbid case of EBV-associated ANKL. Initially isolated from Burkitt’s lymphoma [10], EBV is one of the most potent transforming human viruses in culture due to its ability to infect B cells and induce their proliferation [11]. This oncogenic virus, also known as human herpesvirus 4, is a member of the gamma herpesvirus family and is classified into two strains based on the EBV nuclear antigen sequence: EBV1 and EBV2. EBV1, which is more prevalent worldwide, is associated with more efficient lymphocyte transformation, more severe liver enzyme dysfunction, and a shorter clinical course compared to EBV2, which is more common in Alaska, Papua New Guinea, and Central Africa [2,4].

EBV infects over 90% of the adult population worldwide. It is primarily transmitted through saliva, but other methods include blood transfusion and allograft transplantation. Like all human herpesviruses, EBV has a biphasic life cycle consisting of lytic and latent phases. In the lytic phase, which occurs during the primary infection and viral reactivation, transient productive replication occurs, followed by the lysis of host cells and the release of infectious virions [2].

During the primary infection, EBV targets resting B lymphocytes as well as epithelial cells, although it is unclear which is infected first [2,3]. Primary EBV infection in the pediatric population is often subclinical, with symptoms similar to other respiratory illnesses, while in adults, it commonly manifests as acute infectious mononucleosis [1]. After the initial infection, EBV establishes latency in circulating memory B cells through an unclear mechanism, exhibiting distinct latency patterns [3,12]. In immunocompetent individuals, this latency is asymptomatic and is maintained by immune surveillance by cytotoxic T cells and NK cells. However, disturbances in the host immune system due to oxidative stress, co-infection with other viruses, immunosuppression, and organ transplantation can stimulate viral reactivation [2].

The latency patterns of EBV, which vary based on the proteins expressed, are implicated in EBV-mediated oncogenesis [2]. EBV-related malignancies account for 1.5% of all human cancers [2] and 1.8% of all cancer deaths [13], and are classified as lymphoproliferative, epithelial, or soft tissue in nature [2,3]. These malignancies have geographic and epidemiologic peculiarities. For example, nasopharyngeal carcinoma and Burkitt’s lymphoma are more prevalent in Southeast Asia and Africa, while T/NK cell LPD are more common in East Asia and South America. Considering that EBV is ubiquitous and predominantly causes asymptomatic infection, the peculiar geographic distribution of EBV-associated malignancies suggests that host-specific genetic factors and environmental cues likely facilitate EBV-mediated oncogenesis [3,4].

In rare cases, a high viral load from primary infection or reactivation may result in EBV infection of T and/or NK cells, leading to their dysfunction and/or a reduction in their numbers [14]. Impairment of the immune surveillance mechanism may in turn allow for CAEBV [15]. Clinically, CAEBV of T and/or NK cells is an aggressive systemic disease characterized by infectious mononucleosis-like symptoms persisting for over three months, high serum EBV titer, and histologic evidence of organ infiltration by EBV-positive lymphocytes [5]. In addition to causing end-organ damage, CAEBV may increase the risk of hematologic malignancies like ANKL by causing mutations and inducing the expression of pro-survival factors in infected T and/or NK cells [16,17]. CAEBV is thought to be associated with latency types I and/or II [3,12].

ANKL, which is associated with EBV in 85-100% of cases and latency types I and/or II, predominantly affects young to middle-aged adults and clinically manifests with fever, malaise, hepatosplenomegaly, liver failure, pancytopenia, elevated serum LDH, and variable numbers of leukemic NK cells in the peripheral blood ranging from less than 5% to over 80% of total leukocytes. Occasional lymphadenopathy may also be observed [5]. Complications associated with this condition include HLH, coagulopathy, and multiple organ failure [5]. Among the various EBV-associated LPDs, ANKL carries the worst prognosis, with a median survival of only 1.6 months [18]. Of note, liver dysfunction and late-onset (>eight years old) are associated with increased mortality [19].

In the presented case, the patient developed EBV-positive NK cell predominance in peripheral blood and bone marrow, consistent with ANKL. Re-staining of a liver biopsy obtained prior to the current hospitalization revealed hepatic infiltration by EBV-positive NK cells. Although a serum EBV titer was not obtained during that admission, these findings raise the possibility that the patient may have had undiagnosed CAEBV of undetermined chronicity, which potentiated the development of ANKL. This may have been further exacerbated by the immunosuppression of pregnancy. Alternatively, the patient may have developed ANKL de novo, although EBV positivity in NK cells prior to the onset of malignancy strongly supports ongoing CAEBV as a likely pathogenic driver.

Due to the rarity of T/NK cell LPD, treatment advancements have been limited. In the case of CAEBV, therapeutic options include antiviral agents, immune-modulatory drugs, and chemotherapy, but stem cell transplantation remains the only potentially curative treatment with a three-year survival rate of 65-82% [19,20].

Emerging therapies such as JAK1 and JAK2 inhibitors have shown some potential benefits. Onozawa et al. identified hyperactivation of STAT3 in EBV-infected NK and T cells as a key driver of pro-inflammatory cytokine production, contributing to organ damage observed in CAEBV [21]. While STAT3 inhibition may not be curative, it could mitigate inflammation and facilitate stem cell transplantation. A small retrospective study indicated that ruxolitinib, a JAK1 and JAK2 inhibitor, induced remission with a median of 7.1 weeks without decreasing EBV DNA levels [22]. The prototypic anti-herpes virus agents, acyclovir and its derivative ganciclovir rely on viral thymidine kinase for phosphorylation into cytotoxic nucleoside analogs [23]. However, EBV's thymidine kinase efficiency is notably lower than that of herpes simplex virus [24]. Instead, a virally encoded protein kinase (PK), whose expression is restricted to the lytic phase, plays a crucial role in phosphorylating these prodrugs [25]. Therefore, these agents are ineffective against latent EBV replication in CAEBV. Combining ganciclovir with histone deacetylase inhibitors or proteasome inhibitors, which induce viral PK expression via induction of a lytic program, has been shown to enhance the antiviral effects of ganciclovir [9,26]. Ganciclovir is preferred over acyclovir due to its superior cytotoxicity [27]. Our findings, consistent with previous reports, demonstrated a reduction in viral load with ganciclovir/bortezomib therapy; however, viral titers rebounded upon bortezomib discontinuation, possibly due to latency program reactivation and PK silencing. These findings demonstrate the potential therapeutic value of ganciclovir/bortezomib combination therapy.

For ANKL, management typically involves chemotherapy and stem cell transplantation. Promising therapeutic agents include BCL-2 inhibitors, immune checkpoint inhibitors like PD-1/PD-L1 antibodies, JAK1 and JAK2 inhibitors, and histone deacetylase inhibitors [8,28]. The patient of the present case was planned for stem cell transplant but unfortunately developed multi-organ failure and passed.

## Conclusions

EBV primary infections as well as secondary reactivations may cause various EBV-associated LPD including hematologic malignancies, especially within immunocompromised hosts. ANKL is an exceedingly rare and malignant LPD of NK cell type. ANKL is mostly found in young or middle-aged adults from Asia and South America, either developing on its own or in the setting of other NK cell type LPD (i.e., CAEBV or extranodal NK/T lymphoma). The cornerstone of management of CAEBV and ANKL involves chemotherapy and hematopoietic stem cell transplantation. The role of antiviral therapy is limited as nucleoside analogs have no effect on latent infection. However, it has been hypothesized that an effective strategy would be to combine an antiviral agent with drugs that can induce lytic gene expression in EBV-infected cells. Ganciclovir has been preferred as its phosphorylated form not only inhibits viral replication but is also cytotoxic to infected cells compared to acyclovir.
